# Case Report: Recurrence of recipient-derived erythropoiesis in hematopoietic stem cell transplant for sickle-cell anemia: multi-lineage chimerism analysis as a tool to distinguish atypical pure red cell aplasia from poor erythroid engraftment

**DOI:** 10.3389/fimmu.2026.1932283

**Published:** 2026-09-25

**Authors:** Claire Geurten, Evelyne Willems, Nathalie Dhedin, Corinne Pondarre, Annarita Miccio, Ivan Sloma, Maria Bou Dames, Louise Vrancken, Jaques Foguenne, Jérôme Coupier, Frederic Baron

**Affiliations:** 1 Department of Paediatric Haemato-oncology & Stem Cell Transplantation (SCT), Universitaire Ziekenhuizen Leuven, Leuven, Belgium; 2 Department of Paediatric Haemato-oncology & Stem Cell Transplantation (SCT), Centre Hospitalier Universitaire de Liège, Liège, Belgium; 3 GIGA Immunobiology, University of Liège, Liège, Belgium; 4 Department of Clinical Haematology, Centre Hospitalier Universitaire de Liège, Liège, Belgium; 5 Department of Haematology Adolescent & Young Adults, Hôpital Saint-Louis, Paris, France; 6 Centre Hospitalier Intercommunal de Créteil, INSERM Unité 955, Paris XII Université, Créteil, France; 7 Laboratory of Chromatin and Gene Regulation During Development, Institut Imagine, Institut national de la santé et de la recherche médicale UMR 1163, Université Paris Cité, Paris, France; 8 Department of Haematology and Immunobiology, Institut Mondor de Recherche Biomédicale, Institut national de la santé et de la recherche médicale UMR 955,Assistance Publique - Hôpitaux de Paris (AP-HP) Hôpitaux universitaires Henri Mondor, Paris, France; 9 Department of Biological Haematology, Centre Hospitalier Universitaire de Liège, Liège, Belgium; 10 Department of Genetics, Centre Hospitalier Universitaire de Liège, Liège, Belgium

**Keywords:** case report, chimerism, erythroid precursor, graft rejection, Pure Red Cell Aplasia, sickle cell anemia, stem cell transplant

## Abstract

Experience in hematopoietic stem cell transplant (HSCT) as a long-term curative treatment for sickle-cell anemia (SCA) has been gathered over the last 30 years. Sustained engraftment to restore normal erythropoiesis remains the primary aim of HSCT in this disease. Yet, with the increasing use of reduced intensity conditioning as a toxicity-sparing alternative, a significant proportion of transplanted patients develop mixed chimerism. While a myeloid donor chimerism above 20% is deemed sufficient to prevent pain crisis, patients with donor chimerism below 50% can present recurrence of hemolytic features (1). Although experimental studies have demonstrated the feasibility of chimerism analysis on CD71-positive erythroblasts as well as earlier erythroid precursors such as BFU-E and CFU-GM, their clinical benefit remains debated (2, 3). Herein we report the unusual case of a 15 year-old patient transplanted for SCA who developed Pure Red Cell Aplasia (PRCA), followed by the recurrence of hemolytic anemia and rising HbS levels upon reticulocyte recovery. The patient failed to respond to standard treatment with Rituximab and Daratumumab for PRCA, which further raised concern for poor donor erythroid engraftment or secondary graft failure. In this setting, the information supplied by lineage-specific chimerism, particularly B-cell and erythroid progenitor chimerism, which were both predominantly of donor origin, helped distinguishing the two conditions and guide clinical decision-making. The hematological condition of the patient dramatically improved upon donor-derived B-cell recovery and initiation of plasmapheresis. This clinical report makes an argument for lineage-specific chimerism in patients with hemoglobinopathies experiencing recurrence of hemolytic anemia after PRCA.

## Introduction

Sickle cell anemia (SCA) is the most common inherited blood disease worldwide, affecting about 7.7 million people across the globe in 2021 (1). Curative options had long been limited before the feasibility and efficacy of hematopoietic stem cell transplant (HSCT) were demonstrated following the first matched sibling bone marrow transplantation in a sickle cell patient affected with acute myeloblastic leukemia (AML) in 1984 (2). The procedure subsequently cured the patient from both ailments. Since then, HSCT in hemoglobinopathies has traveled a long way, gradually evolving from myeloablative conditionings to reduced intensity conditionings (RIC) intended to reduce treatment-related toxicity, notably on fertility (3). Yet, Conditioning reduction is associated with a higher incidence of mixed chimerism, the management of which present different challenges (4). Indeed, dropping chimerism can evolve to recurrence of SCA-related events. Despite demonstration in case reports that donor lymphocyte infusion (DLI) could eradicate recurrent host hematopoiesis after HSCT for SCD (5) the procedure expose patients to a high risk of graft-versus-host-disease which is often deemed too risky in this setting.

It is generally accepted that a full donor chimerism is not required to cure SCA and cure has been reported following allogeneic bone marrow transplantation with heterozygous carrier donor (6). Indeed, case studies have confirmed that HSCT recipients formerly affected with SCA could achieve event-free remission with a whole blood chimerism as low as 20%, and hemolysis-free remission provided donor chimerism was above 50% (7, 8). It is hypothesized that the ability of a low donor whole blood chimerism to reverse clinical phenotype stems from a survival advantage exhibited by healthy donor erythrocytes over sickle red blood cells, resulting in a favorable erythroid chimerism, irrespective of whole blood chimerism (9).

Experimental studies focusing on extensive erythroid chimerism analysis have studied the ability of chimerism in CD71-positive erythroblastic cells, Colony-Forming-Unit Granulocyte-Macrophage (CFU-GM) and Burst-Forming-Unit-Erythrocyte (BFU-E) to predict impending graft failure (9). These studies have demonstrated the feasibility of the assays based on bone marrow samples, although the information provided seemed to match that of whole blood donor chimerism, meaning the clinical impact of erythroid precursor chimerism is limited and does not compare favorably to the more accessible whole blood chimerism (10–12). Redundant information would not warrant a relatively invasive bone marrow puncture in those patients, who would otherwise only be monitored based on blood samples. Yet, the fact that patients transplanted for SCA experience disease remission with low level of whole blood donor chimerism suggests that mature erythroid chimerism does not evolve in parallel to whole blood chimerism in this specific population. The preliminary studies on CFU-GM, CD15+, and BFU-E chimerism in SCA patients with mixed chimerism (donor chimerism <70%) demonstrated a progressive enrichment of donor-derived cells during erythroid maturation, highlighting the survival advantage of healthy erythrocytes over their sickle counterparts (11). This suggests that the real transplant outcome may be more accurately assessed by measuring donor HbA levels than by evaluating erythroid precursor or myeloid chimerism.

As such, up to date, erythroid chimerism in clinical practice is mostly estimated on the basis of the blood group and hemoglobin electrophoresis, provided the patient has remained transfusion-free, which is unfortunately often not the case in the first months after HSCT. Some authors have advocated that myeloid chimerism could be substituted to whole blood chimerism to predict evolution to secondary graft failure and SCA recurrence, thereby requiring clinical intervention such as adaptation of immune suppressive therapy or DLIs. There is, however, little evidence to support this that decreasing myeloid chimerism is a better marker of upcoming SCA recurrence than dropping whole blood chimerism (3, 10–12).

## Case description

Here, we report the case of a 15 year-old girl with homozygous SCA who received a peripheral blood stem cells allograft (CD34 9.21x10e6/kg) from her HLA-matched sister, who carried a sickle cell trait (HbAS, with baseline HbS 44%), after reduced intensity conditioning, consisting in alemtuzumab (0.03 mg/kg on day −7, 0.1 mg/kg on day −6, and 0.3 mg/kg/day on days −5 to −3) and 3 Gy total body irradiation (TBI) on day −2. Sirolimus-based GVHD prophylaxis was started on day -1. Both donor and recipient were CMV positive. There was a major ABO mismatch (recipient O+ and donor A+) with high recipient pre-transplant anti-A titers, measured at 1/256. The patient had been prepared to HSCT with monthly erythrocytapheresis for 6 months before HSCT; and HbS level on admission to the transplant unit was <20%. The early transplant course was uncomplicated except for an early herpes zoster infection treated with ganciclovir followed by maintenance and secondary prophylaxis with valaciclovir. Although platelets and neutrophils engrafted by day 14, the patient maintained transfusion-dependent with low reticulocyte count (7000-15.000/mm3) and high anti-A titers of 1/256, in line with a diagnosis of Pure Red Cell Aplasia (PRCA). Donor whole blood chimerism at day 40 was 98.7%, and 90.8%, at day 100. T-cell donor chimerism at day 100 was low at 38.3%, as expected following alemtuzumab-based HSCT. The evolution of hematological parameters, chimerism and transfusion-dependency is illustrated in **Figure 1**. Transfusions were administered at an interval of 10–14 days to maintain hemoglobin above 9 g/dL during the peri-engraftment period. Four months post-transplant, we observed a progressive rise in reticulocyte count, initially around 180.000/mm3 then up to 437.000/mm3. Hemolysis parameters progressively increased with a concomitant concerning rise in HbS levels (up to 56.3% at day 180, whereas the donor baseline HbS level was 44%), suggesting graft rejection or poor erythroid engraftment. Yet, at this time, donor whole blood chimerism, myeloid and T-cell chimerism were respectively 84.4%, 83.3% and 57%. In light of this information, we hypothesized that recovery of recipient’s reticulocytes stemmed from a suppression of donor erythropoiesis driven by recipient’s anti-A antibodies. We proceeded to administer treatment for PRCA with 4 courses of Rituximab 375 mg/m2 which failed to demonstrate any benefit at 8 weeks, followed by Daratumumab 16 mg/kg SC administered weekly for 4 weeks. There was no impact on anti-A titers (1/256) or HbS (63.7%), although transfusions could progressively be spaced out to a monthly interval as hemoglobin stabilized above 9 g/dL. Still, hemoglobin at 4 weeks post-treatment remained low at 8.3 g/L with high reticulocytes (367.000/mm3). At 12 months post-transplant, i.e. 14 weeks since initiation of Daratumumab, the complete blood count was unchanged, red blood cells remained exclusively of recipient’s or transfused origin (O+) while anti-A titers decreased to 1/8. In this context of unsatisfactory response to Daratumomab at more than 3 months since completion of treatment, we initiated plasmapheresis, and after three sessions, anti-A titers were successfully decreased to 1/1 and A+ red-blood cells were identified in peripheral blood (**Figure 2**). The patient had received her last blood transfusion 3 months before initiation of plasmapheresis and remained transfusion-independent afterwards.

**Figure 1:**
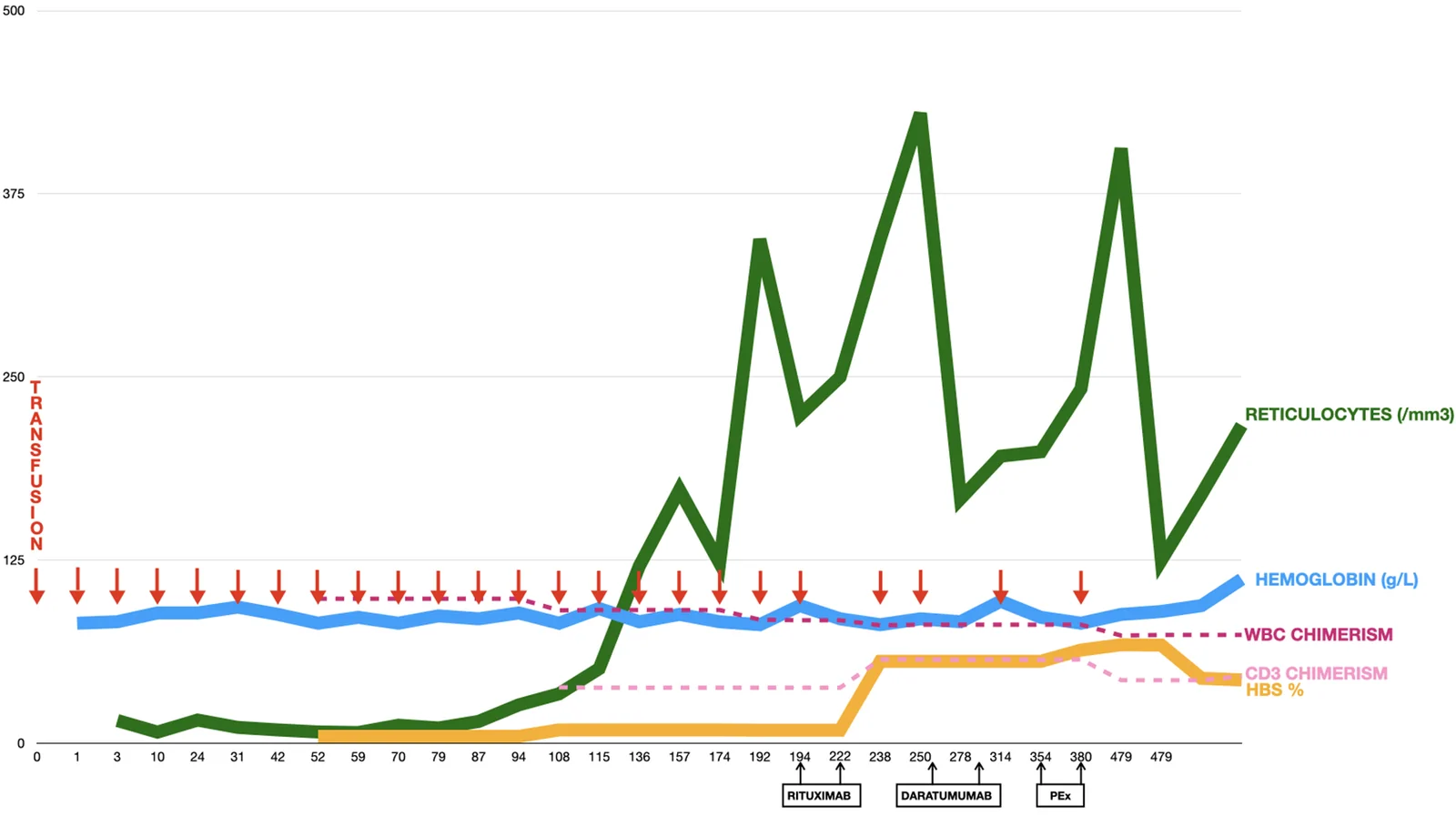
Evolution of whole blood and lineage-specific chimerism, HbS percentages according to time elapsed since HSCT and treatment received. Transfusions (red arrows) were initially administered at an interval of 10–14 days to maintain hemoglobin above 9 g/dL. At four months post transplant the patient presented persistent anemia with a sudden rise in reticulocyte count, as well as other hemolytic features. We observed mixed WBC donor chimerism (84.4%), CD3 chimerism (38%). Rituximab was initiated around day 194 due to persisting high anti-A titers but did not improve the hematological condition. When transfusions were spaced out, a concerning rise in HbS levels raising suspicion for poor erythroid engraftment. Treatment with Daratumumab from day 262 to 290 had no impact on hemoglobin and HbS levels. WBC chimerism remained above 50% (74% at 1 year post transplant). CD33 and CD19 chimerism were both predominantly donor at this stage, respectively 83% and 79%. The complete blood count normalized after initiation of plasmapheresis.

**Figure 2:**
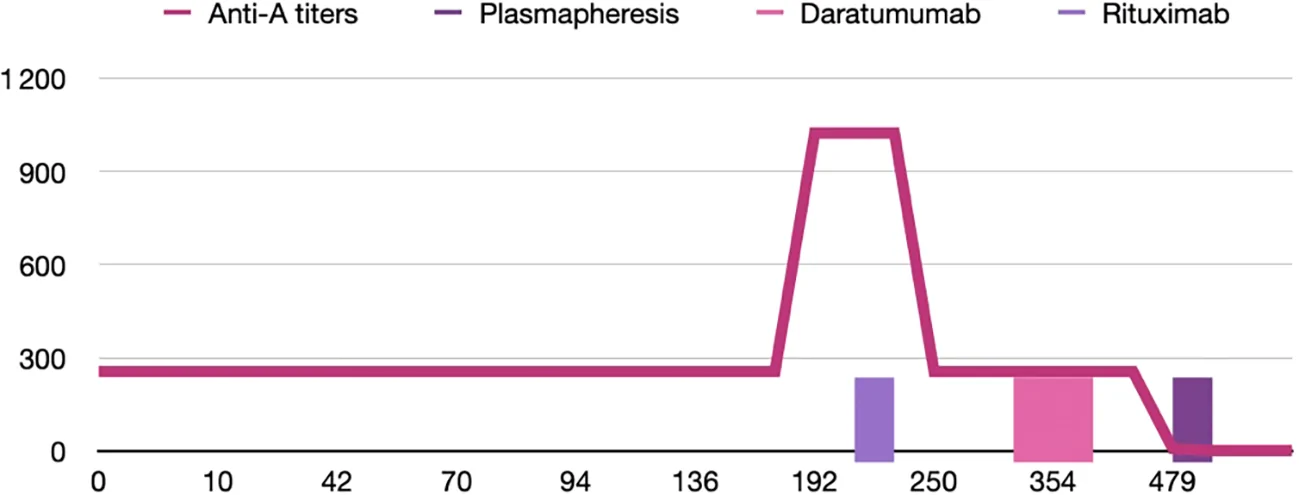
Evolution of anti-A titers according to time elapsed since HSCT and treatment received. Administration of Rituximab did not result in a reduction of anti-A titers below the threshold of clinical significance, and no positive impact of Daratumumab treatment was noted in the first nine weeks following therapy. On day 479, when plasmapheresis were initiated, anti-A antibodies had faltered. Our hypothesis is that B-cell chimerism upon recovery from treatment with B-cell targeting therapies became predominantly donor and that anti-A antibody production was halted, enabling hematological recovery.

In order to confirm the absence of graft rejection and assess the safety of plasmapheresis interruption, we performed a bone marrow aspiration at 14 months post-transplant to extensively study the erythroid chimerism. Bone marrow mononuclear cells were first isolated by density gradient centrifugation over Lymphocytes Separation Medium (MSL^®^, Eurobio Scientific; density 1.078 g/mL), and CD34+ multipotent hematopoietic stem/progenitor cells (HSPCs) were subsequently purified from the mononuclear fraction by immunomagnetic selection with MACS columns (Miltenyi Biotec) after immunostaining with the CD34 MicroBead Kit (Miltenyi Biotec). Purified CD34+ HSPCs were plated at a concentration of 250 cells/mL in a methylcellulose-based medium (GFH4435, Stem Cell Technologies) under conditions supporting the growth and differentiation of committed erythroid (Burst-Forming Unit-Erythrocytes, BFU-E) and granulo-monocytic (Colony-Forming Unit-Granulocytes Macrophages, CFU-GM) progenitors and incubated at 37 °C in a humidified atmosphere with 5% CO2. BFU-E and CFU-GM colonies were picked after 14 days of culture based on standard morphological criteria, with BFU-E identified as dense, hemoglobinized clusters of red/pink erythroblasts and CFU-GM identified as more diffuse, pale colonies of granulocytic and monocytic cells. 24 single BFU-E and CFU-GM colonies were then individually picked under an Olympus stereomicroscope, in order to resolve donor/recipient chimerism at the level of individual clonogenic progenitors. Afterwards, DNA was extracted and chimerism analysis was performed. All BFU-E colonies, and 80% of CFU-GM colonies were from donor origin (**Figure 3**). In parallel, chimerism analyses on isolated CD34+ cells and CD71+ erythroid precursors from the bone marrow, showed that HSPCs and erythroid precursors were predominantly of donor origin at this stage (87% and 82.6%, respectively). CD19+ B-cell donor chimerism in peripheral blood was 78.9%. These results were consistent with the clinical evolution of the patient who on the day of the bone marrow aspiration exhibited a HbS level of about 44%, comparable to that of her donor. The patient was no longer anemic, reticulocyte count stabilized around 170.000/mm3 with no further sign of hemolysis. At 22 months post-transplant the patient has been successfully weaned from sirolimus for the last six months, has recovered normal CD4 and CD19 counts. There persists a mild anemia (Hb 11.1 g/dl) without microcytosis, and a HbS of 41.6%, consistent with the donor’s hematological status.

**Figure 3:**
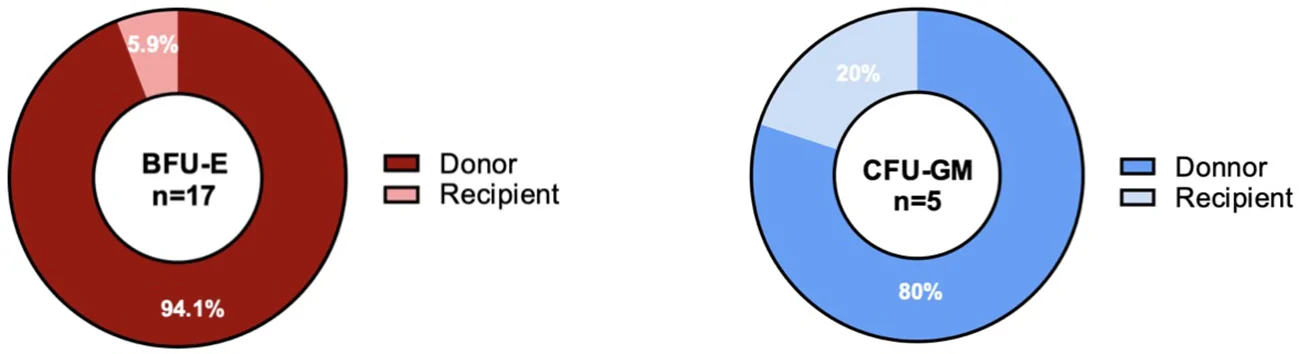
Bone marrow progenitor chimerism 14 months post-allograft. Peripheral blood DNA from both the donor and the recipient, collected before the allograft, were genotyped with KMR Type (DenDx^®^) and analyzed using KMR Engine software (GenDx^®^) according to the manufacturer’s instructions. The markers KMR040 and KMR043 were specific to the recipient and the donor, respectively, and were therefore selected to genotype the BFU-E and CFU-GM colonies in order to identify the progenitor’s origin. CD34^+^ cells were purified from patient’s bone marrow using the CD34 MicroBead Kit (human) and plated at a concentration of 250 cells/mL in a methylcellulose-based medium with recombinant cytokines (GFH4435, Stem Cell Technologies) under conditions supporting erythroid and granulocyte/monocyte differentiation. We obtained 44 BFU-E colonies and 24 CFU-GM from 1,500 CD34^+^ HSPCs. After 14 days, colonies were scored and collected individually (24 individual BFU-E and CFU-GM colonies, respectively). Individual colonies were genotyped by qPCR using KMRtype (GenDx). For each colony, both markers were tested alongside an internal control. Colonies that had insufficient internal control or displayed a mixed profile, with detection of both donor and recipient markers (Ct<40), were excluded from the analysis. The qPCR results were interpretable for 17 out of 24 BFU-Es and 5 out of 8 CFU-GMs tested.

## Discussion

Pure Red Cell Aplasia (PRCA) is a well described complication of ABO-mismatched HSCT, characterized by persisting transfusion dependency with low reticulocyte count for up to 60 days after HSCT. It is diagnosed in about 10-15% of HSCT and is commonly associated with high pre-transplant isohemagglutinins titers in the context of major ABO mismatch (13). Anti-A isoagglutinins tend to wane slower than anti-B (28 vs 16 days) (14), explaining why most PRCA cases occur in the following transplant of a group A recipient with a group O donor. Additional risk factors appear to be sibling donor and reduced-intensity conditioning (13). A cut-off of isohemagglutinins as high as 1/64 has been suggested to deliver 100% sensitivity and 68.8% specificity for PRCA diagnosis (13). Treatment of PRCA relies on transfusions and is less likely to spontaneously resolve after 2 months of evolution, thereby requiring additional immunosuppressive therapy such as rituximab, daratumumab, and more rarely, plasmapheresis.

This report represents the first description of a case of atypical PRCA in the context of ABO mismatched HSCT for SCA, characterized by recurrence of recipient-derived reticulocytosis and hemolytic features at day 120 as illustrated by hemoglobin electrophoresis and blood group, due to suppression of donor-derived erythropoiesis by anti-A antibodies. Whole blood and myeloid donor chimerism remained above 80% suggesting sufficient engraftment, although mixed chimerism in the context of reduced intensity conditioning allowed for co-existence of erythroid precursors from both donor and recipients, enabling recipient-derived erythropoiesis to take over. The remanence of anti-A titers suggested an immune-mediated silencing of donor-derived erythropoieis but the patient did not demonstrate any response to anti-CD20 or anti-CD38 directed therapies three months after completing both treatments. The patient’s hematological condition improved dramatically with anti-A titers decrease, as illustrated by the rise in hemoglobin level with simultaneous decrease in HbS levels. Extensive chimerism study of erythroid progenitors in the bone marrow performed 4 weeks after interruption of plasmapheresis showed these cell types were predominantly donor-derived, although with a mixed chimerism <95%. B-cells had partially recovered at the time of testing and were predominantly of donor origin too at this stage, which was consistent with the improvement of the patient’s hemolysis parameters and the decrease in anti-A titers.

The severity of the anemia and the anti-A titers had started to decrease before initiation of plasmapheresis, suggesting at least a partial delayed effect of Daratumumab through disappearance of antibody-producing recipient CD20^neg^CD38^low^ plasma cells in the bone marrow. Still, the literature reports a mean time to response after daratumumab of 28–60 days (13, 15–20), which is much shorter than the 92 days that had elapsed before anti-A titers started to decrease in our patient. This highlights the fact that plasmapheresis still holds a place in the therapeutic arsenal for PRCA, and that lineage-specific chimerism explorations can represent an added value in atypical case with unexpected lack or delayed response to treatment, specifically when the desired outcome of HSCT is resolution of a red blood disorder.

It is likely that progressive evolution towards a prominent donor B-cell chimerism contributed to clinical resolution of PRCA and resurgence of SCA in this patient. Conversion of B-cell chimerism could have been delayed by our choice to use B-cell targeting therapies as treatment for PRCA. We could hypothesize that an infusion of DLIs, carefully pondered due to the inherent risk of GVHD, could have brought a low but sufficient number of donor B-cells to help convert B-cell chimerism and reverse the sickling phenotype earlier, but, having chosen a different therapeutic approach, we are unable to confirm this hypothesis.

Unfortunately, we did not obtain bone marrow samples to assess erythroid precursors chimerism nor have we obtained documentation of donor B-cell chimerism at earlier timepoints in the evolution of our patient’s course. The evolution of these parameters would have been highly informative, particularly chimerism on BFU-E colonies. PRCA typically affects CFU-GM and erythroid precursors, whilst BFU-E units are preserved (20). It is thus likely that all CFU-GM colonies in the earlier course of PRCA would have been of recipient’s origin, whereas BFU-E chimerism would have helped to confirm erythroid donor engraftment. As bone marrow aspirations are not routinely performed in follow-up for hemoglobinopathies and can be perceived as an invasive procedure when conducted for scientific purposes only, we performed the extensive multi-lineage chimerism analysis at a timepoint where it was felt warranted as the results affected management, namely discontinuation of plasmapheresis, and withdrawal of immune suppressive therapy.

Although available literature suggests pre-HSCT preventive removal of elevated anti-A titers with plasmapheresis fails to prevent occurrence of PRCA (13), interesting results have been described with pre-HSCT Rituximab (21, 22), and this preventive option should definitely be pondered in major ABO-mismatched transplant with high pre-HSCT recipient anti-A titers, especially in the context of reduced-intensity conditioning, where multilineage mixed chimerism can lead to an atypical evolution of PRCA where recipient-derived plasmocytes hamper the settlement of donor-derived erythropoiesis due to continued exposure to anti-A antibodies, allowing recipient-derived erythropoiesis to take over and lead to resurgence of SCA.

In such cases, our observation suggests that PRCA may adopt an unusual course following reduced-intensity conditioning in HSCT for SCA, particularly in the context of major ABO-mismatched transplant. In the setting of mixed chimerism, the coexistence of donor- and recipient-derived cell lineages can result in recurrence of hemolytic anemia and raise concern for impending graft failure, based on rising HbS levels. Assessment of erythroid progenitors chimerism is a useful tool to distinguish between inadequate erythroid donor engraftment and predominance of recipient-derived erythropoiesis due to selective suppression of donor-derived erythroid precursors at a later stage of maturation by anti-ABO alloantibodies, thereby meaningfully informing clinical management.

## Patient’s perspective

The comprehensive diagnostic work-up performed in this patient enabled a definitive diagnosis and allowed physicians to reassure the patient regarding the outcome of her hematopoietic stem cell transplant. The initiation of plasmapharesis normalized her hematological condition and the extensive chimerism analysis confirmed a low risk of recurrence. These findings allowed physicians to proceed to the weaning of immunosuppressive therapy, ultimately allowing the patient to resume a normal life, now sickle-free.

## Data Availability

The original contributions presented in the study are included in the article/supplementary material. Further inquiries can be directed to the corresponding author.
